# Central Surgeries

**Published:** 1923-03

**Authors:** 


### CORRESPONDENCE.

[Correspondence on all subjects is invited, but we cannot be responsible for the opinions expressed by our correspondents, who must give name and address as a guarantee of good faith, but not necessarily for publication. Correspondents are reminded that conciseness greatly facilitates early insertion.]

### Central Surgeries.

*To the Editor of* The Hospital and Health Review.

Sir,—I am with you all the way in the suggestion for establishment of Central Surgeries made in your article last month on "The Organisation of Panel Practice," and believe this to be one of the lines on which general practice must develop if it is to keep abreast of the advances of science. My experience at the out-patient department of a large general hospital during the war brought home to me the very great advantages both to the patient and the doctor. Indeed, I put out certain "feelers" two or three years ago, but they were not, to say the least, very enthusiastically received.

For one thing, the housing difficulty is at present an insuperable bar and likely to be with us, at any rate, for some time. In a place of this size and geographical distribution we should require six or seven Central Surgeries, and I am of opinion that a start might be best made in a small town of some 60,000 or 80,000 people, where one central surgery might quite efficiently meet the case. There is not only the innate conservatism of the profession to break down, but, even more, in this part of the country that of the patients, who will go to the same old place no matter what the conditions may be.

A Busy General Practitioner.

